# *Streptococcus pneumoniae*, an unusual cause of early‐onset neonatal sepsis and necrotizing pneumonia

**DOI:** 10.1002/ccr3.1640

**Published:** 2018-07-02

**Authors:** Miren Alicia Apilánez Urquiola, Olaia Sardón Prado, Javier Korta Murua, Paula Corcuera Elosegui, Miguel Ángel Cortajarena

**Affiliations:** ^1^ Division of Neonatology Donostia University Hospital San Sebastián Spain; ^2^ Division of Pediatric Respiratory Medicine Donostia University Hospital San Sebastian Spain; ^3^ Department of Pediatrics University of the Basque Country (UPV/EHU) San Sebastian Spain

**Keywords:** infectious diseases, respiratory medicine

## Abstract

Vertically transmitted sepsis due to *Streptococcus pneumoniae* has a low incidence, and vaginal colonization among pregnant women is exceptional. Necrotizing pneumonia is uncommon in immunocompetent term neonates, and the prognosis is uncertain. At present, systematic screening does not seem warranted in pregnant women. Therefore, aggressive treatment of neonates remains the best treatment.

## 1 INTRODUCTION

Vertically transmitted sepsis due to *Streptococcus pneumoniae* has a low incidence. Vaginal colonization is exceptional. We report the case of an immunocompetent term neonate with early‐onset pneumococcal sepsis and bilateral necrotizing pneumonia. Systematic screening does not seem warranted in pregnant women. Therefore, aggressive treatment of neonates is the best therapeutic strategy.

Vertically transmitted neonatal sepsis due to *S. pneumoniae* is relatively rare and has an incidence of approximately 1%‐11% of cases of neonatal sepsis.^1^ However, according to the available data, the associated morbidity and mortality is high (14.3%‐60%), causing mainly neonatal sepsis, early‐onset bacterial pneumonia(<72 hours), and meningitis.^1^, ^2^ It has been associated with prolonged rupture of the membranes, vaginal colonization in pregnant women (0.03%‐0.75%), chorioamnionitis, pneumonia, and maternal meningitis.^2^ We report a case of early‐onset pneumococcal sepsis and bilateral necrotizing pneumonia in an immunocompetent term neonate.

## 2 CASE REPORT

The patient was a term neonate (38 + 3 weeks of gestation), with adequate birthweight for gestational age. Prenatal ultrasound findings were normal. The pregnancy was the mother’s fourth, with good prenatal care. The results of maternal serum tests and vaginal swab culture were negative for *Streptococcus agalactiae*. The mother had prolonged rupture of the amniotic sac (24 hours), prompting induction of labor, which was uneventful, with spontaneous vaginal delivery. The mother had puerperal fever (37.9°C). No new vaginal cultures were taken.

At 30 hours of life, the newborn was admitted to the Neonatal Unit from the Maternity Unit, due to respiratory grunting, discoloration of the skin and peripheral hypoperfusion, raising clinical suspicion of sepsis. Complementary tests: leukopenia and thrombocytopenia, with normal red blood cell and coagulation tests; metabolic acidosis; C‐reactive protein 255 mg/L and procalcitonin 22 ng/mL. Cerebral spinal fluid and urine analysis were normal (Table 1). The admission chest X‐ray showed bilateral basal alveolar infiltrate**.** Respiratory support was initiated with high‐flow oxygen therapy and volume expansion, and empirical antibiotic therapy was started with ampicillin (150 mg/kg/d) and cefotaxime (200 mg/kg/d). Afterward, the patient showed clinical deterioration, with fever of up to 39°C and involvement of general status, requiring admission to the Neonatal Intensive Care Unit (NICU), respiratory support (high‐flow oxygen therapy) and vasoactive support (dopamine). On the third day of admission, a positive blood culture result for cefotaxime‐sensitive, ampicillin‐sensitive and gentamicin‐resistant *S. pneumoniae* (serotype 12F) was received. Therefore, according to the minimum inhibitory concentrations received (MIC 0.015 for cefotaxime and 0.023 for ampicillin), the spectrum of the two antibiotics (higher for the cefotaxime) and the favorable clinical evolution presented by the patient (72 hours of antibiotic therapy started), the treatment was continued only with intravenous cefotaxime (200 mg/kg/d). The remaining cultures were negative. Study of immunodeficiencies, screening for cystic fibrosis, sweat test, and ear and eye swab culture were negative. The results of echocardiography and ECG were normal.

**Table 1:** Laboratory test results

Blood	
Hemogram	
White cell (count/μL)	2760 (9000‐34 000)
Neurocytes (count/μL)	1520 (3300‐22 500)
Platelet (count/μL)	50 000 (220 000‐490 000)
Hemoglobin (g/dL)	18.4 (14.5‐23)
Hematocrit (%)	52.2 (43.5‐70)
Gasometry	
Ph	7.26 (7.2‐7.41)
HCO3 (mmol/L)	18.8 (21‐28)
Base excess (mmol/L)	−8.3 (−2 to 3)
Coagulation time	
Prothrombin time (%)	43 (70‐140)
Prothrombin (I.N.R)	1.83 (0.85‐1.2)
APTT (seg)	38.8 (24‐36)
APTT (Ratio)	1.32 (0.8‐1.2)
C‐reactive protein (mg/dL)	255 (0‐5)
Procalcitonin (ng/mL)	22 (<0.5)
Blood culture	*Streptococcus Pneumoniae*
Urine	
Urine culture	Negative
Cerebrospinal Fluid (CSF)	
Erythrocytes (cells/mm^3^)	36 700
Leukocytes (cells/mm^3^)	24 (0‐5)
Glucose (mg/dL)	61 (40‐70)
Proteins (mg/dL)	117 (15‐40)
CSF culture	Negative

The normal values were provided between brackets.

APTT, activated partial thromboplastin time; I.N.R, international normalized ratio.

**Figure 1:**
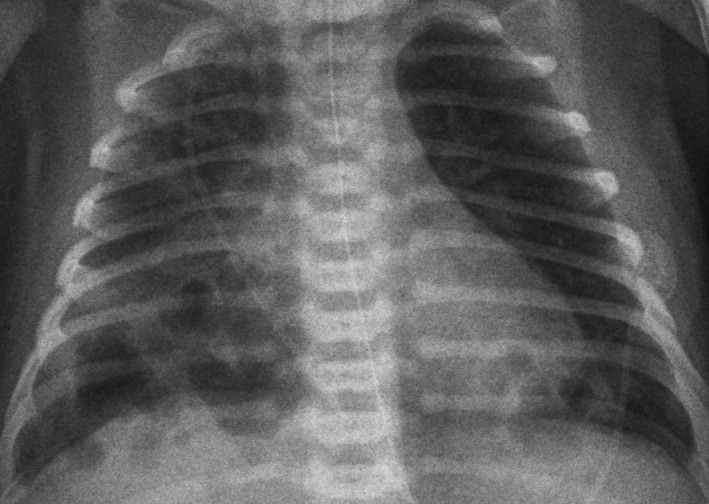
Thorax radiography. Bilateral basal condensation. Bilateral cystic thick‐walled cystic images within the condensation

**Figure 2:**
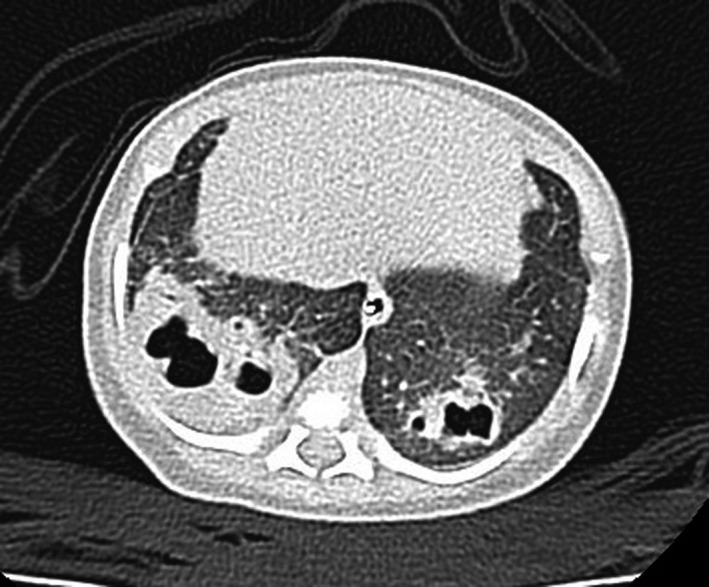
Pulmonary CT scan. Multiple thick‐walled cystic images within bilateral basal condensation and in the right upper lobe and pneumatoceles in the context of necrotizing pneumonia

At first, the patient’s general status improved rapidly with a decrease of acute‐phase reactants but with persistence of signs of respiratory distress with a slowly favorable clinical course, which finally allowed withdrawal of respiratory support. However, on the 10th day of admission, the patient showed respiratory deterioration, with recurrence of respiratory grunting and polypnea, and persistence of bilateral basal condensation. Chest X‐ray revealed multiple thick‐walled cystic images within the condensation and in the right upper lobe (Figure 1). Given the suspicion of bilateral necrotizing pneumonia, a chest CT scan was performed, confirming the diagnosis (Figure 2). Afterward, treatment with intravenous vancomycin (30 mg/kg/d) was added. The clinical course was favorable, allowing the patient to be discharged at 28 days of life with normal physical examination (26 days of cefotaxime and 18 days of vancomycin were completed).

## 3 DISCUSSION

*Streptococcus pneumoniae* is one of the microorganisms causing highest morbidity in childhood in the respiratory system and otorhinolaryngology region, with invasive disease being the most serious complication.^2^ There are more than 90 serotypes and a higher prevalence of invasive disease has been reported in children younger than 2 years.^2^ In the neonatal period, this form of presentation (pneumonia, sepsis, meningitis) is uncommon,^1^ and has two routes of transmission: vertical, due to vaginal colonization, and horizontal.

As with other microorganisms, there are two forms of presentation of invasive disease in neonates: early, with onset in the first 72 hours of life, and late, developing after the second week of life. Most reported cases of neonatal pneumococcal sepsis have been early onset with symptoms appearing in the first 48 hours after delivery^2^ indicating probable infection during the intrapartum or prenatal period, unlike the series reported by Hoffman et al^1^ in which the mean age of presentation was 18.1 days of life. Low birthweight (below 2.000 g) and rupture of membranes lasting longer than 8 hours have been described as risk factors for neonatal pneumococcal infection.^2^ Among the numerous *S. pneumoniae* serotypes described, perinatal invasive disease has been mainly reported in types 1 to 12, 14, 17 to 19, 23, 27, 28, 31 and 39. However, according to published data,^1^ neonatal mortality seems not to be related to serotype or antibiotic sensitivity. Other authors^3^ described a stronger association between serotypes 3 and 19A and necrotizing pneumonia. In our case, the serotype was 12F, an emerging serotype, not included in the 13‐valent conjugate pneumococcal vaccine.

Our setting is a tertiary hospital with 686 admissions per year to the Neonatal Unit. The hospital serves as a referral center for neonatal care, with 6392 deliveries per year. Our case was the only case of early‐onset neonatal pneumococcal sepsis and bilateral necrotizing pneumonia (incidence of 0.01%).

One of the limitations of this study is the lack of maternal vaginal cultures. However, given the early presentation of symptoms, neonatal infection probably occurred during delivery in the context of previous vaginal colonization.

Necrotizing pneumonia is uncommon during the neonatal period and in immunocompetent term neonates, and therefore, prognosis is uncertain in this age‐group. The most frequently reported pathogens after the first month of life are *S. pneumoniae* and *Staphylococcus aureus*. In agreement with published data on the outcomes of young children with this illness,^4^ our patient showed a favorable response with complete clinical and radiological resolution at 6 months of age.

Due to the low incidence of neonatal *S. pneumoniae* sepsis and the low prevalence of maternal vaginal colonization, systematic screening does not seem warranted in pregnant women, although it should be considered in pregnant women with a history of invasive disease due to the same microorganism.^5^ To the same degree, there are insufficient data to state that immunization of pregnant women with the 23‐valent pneumococcal vaccine prevents infection in infants younger than 3 months. Therefore, aggressive treatment of neonates currently remains the best therapeutic option.

## AUTHORSHIP

MAAU and OSP: were involved in planning and wrote the final manuscript with support from PCE. JKM and MÁC: directed and supervised the work. All authors: discussed and contributed to the final manuscript.

## CONFLICT OF INTEREST

None declared.
